# Pregnancy Concerns as Predictors of Sleep Quality in Primigravid Women: A Cross-Sectional Study

**DOI:** 10.7759/cureus.55442

**Published:** 2024-03-03

**Authors:** Fahimeh Monirian, Farzaneh Soltani, Saman Samavati, Soodabeh Aghababaei, Lili Tapak

**Affiliations:** 1 Family Medicine, Hamadan University of Medical Sciences, Hamadan, IRN; 2 Mother and Child Care Research Center, Hamadan University of Medical Sciences, Hamadan, IRN; 3 Department of Biostatistics, School of Public Health, Hamadan University of Medical Sciences, Hamadan, IRN

**Keywords:** iran, primigravid women, mental health, sleep, pregnancy

## Abstract

Introduction

Poor sleep quality may be a risk factor for adverse pregnancy outcomes. Identifying the predictors of sleep disorders can help design effective interventions. The present study aimed to evaluate the impact of pregnancy concerns on the sleep quality of primigravid women.

Methods

This cross-sectional study was conducted on 220 primigravid women referred to health centers in Hamadan, Iran. In addition to completing the demographic characteristics questionnaire, the Parkinson's Disease Questionnaire (PDQ) questionnaire was completed to determine the level of pregnancy concerns, and the Pittsburgh Sleep Quality Index (PSQI) questionnaire was completed to determine the quality of sleep by pregnant women. Data were analyzed using descriptive statistics, the Pearson correlation coefficient, independent t-test, and univariate and multivariate logistic regression models with a backward method at a 95% confidence level.

Results

Pregnant women's total PSQI score was 13.8 ± 3.08, and with a PSQI ≥ 5 as a cut-off point, 76.3% of the study's participants had poor sleep quality. There was a significant direct correlation between the total score of PDQ and its subscales with the total PSQI score (P < 0.05). The adjusted regression logistic model showed a significant relationship between the PDQ scores, women's educational level (adjusted odds ratio (AOR) 1.19; confidence interval (CI) 95%: 0.19-2.18), and their spouse's educational level (AOR 1.03; CI 95%: 0.02-2.03) with sleep quality scores.

Conclusion

Pregnancy concerns, including concerns about birth and the baby, concerns about physical symptoms and body image, and concerns about emotions and relationships, can reduce the sleep quality of primigravid women. Designing and implementing effective interventions to reduce or modify these common concerns can improve the sleep quality of pregnant women and prevent the adverse effects of poor sleep quality on pregnancy outcomes.

## Introduction

Sleep and related disorders are important factors affecting women's health and well-being [1]. Meanwhile, pregnancy can disrupt sleep patterns and cause sleep disorders due to systemic changes caused by hormonal, mental, emotional, and physical factors [2]. Poor sleep quality is one of the most common problems experienced by 97% of women during pregnancy [3]. In late pregnancy, poor sleep quality, difficulty sleeping, night awakenings, and waking up early increase compared to mid-pregnancy [4]. A decrease in sleep duration may be a risk factor for adverse pregnancy outcomes, such as gestational diabetes, gestational hypertension, and cesarean delivery [5,6]. It may also increase the risk of postpartum depression, which is associated with consequences, such as impaired mother-infant attachment and effective care, as well as behavioral or emotional problems in the infant [7]. In a meta-analysis study, Wang and Jin found that a decrease in sleep duration and quality was associated with an increased risk of preterm delivery. In addition, they identified a significant association between sleep disorders and perinatal depression [8]. Considering the poor outcomes of pregnancy in women with low sleep quality, conducting research on factors related to women's sleep quality during pregnancy is of particular importance.

There have been numerous studies on the factors related to sleep disorders in pregnant women [9,10]. Osnes et al. found that depression and stress were associated with poor sleep quality in Ethiopian pregnant women [11]. Moreover, in a longitudinal cohort study, the researchers found that insomnia during pregnancy could be a sign of mood disorders [12]. Meanwhile, pregnancy concerns are defined as physiological stress that pregnant women experience. Pregnancy-specific stress includes fears and concerns relating to their health and the fetus, physical symptoms, alterations in body shape, adequate physical activity, connections with others, and labor and delivery conditions [13]. Many researchers believe that anxiety is common to all pregnancies, and pregnant women may be persistently concerned about fetal health, care quality, and lifestyle or fear of labor pains. Hormonal changes and negative self-imaging can lead to sexual dysfunction and marital problems, which in itself can be a source of maternal anxiety [14]. Furthermore, the stress of adjusting to a new role as a mother in first-time pregnancy may exacerbate this anxiety and stress. As a result, they experienced doubt due to their inexperience in accepting the responsibility of pregnancy and motherhood [15].

A study in Tabriz City, Iran, showed that pregnancy concerns can predict pregnant women's general health [16]. Given that sleep disorders can harm a mother's health, quality of life, and social interactions and have a negative impact on the fetus and infant, identifying the predictors of these disorders can help design effective interventions. In Iran, various qualitative and quantitative studies on the mental health problems of pregnant women have been conducted. Furthermore, some researchers have studied maternal sleep quality, related factors, and the prevalence of sleep disorders in pregnancy [16-19]. However, to our knowledge, there is no study on the correlation between pregnancy concerns and sleep quality in pregnant women. Therefore, the present study aimed to evaluate the impact of pregnancy concerns on the sleep quality of primigravid women.

## Materials and methods

Study design and participants

This cross-sectional study was conducted on 220 primigravid women referred to health centers in Hamadan, Iran, between May and July 2021. Hamadan City, the capital of Hamadan Province, is located in the west of Iran and is one of the oldest cities in Iran and one of the oldest cities in the world. It has a population of about 554,406, considered one of Iran's coldest cities. Hamadan City has 21 urban health centers that provide outpatient treatment, family health, vaccination, oral and dental health services, and more. In the structure of Iran's primary health care (PHC) system, the care of pregnant women is integrated into the PHC system, and these services are provided in health centers through midwives, following the level of services and the referral system.

The inclusion criteria were normal singleton pregnancy, gestational age of 28-38 weeks, lack of history of infertility, medical or obstetrical complications according to the person's health record, and lack of history of sleep disorders before pregnancy. If the questionnaire was distorted, the participant's answers were vague, or more than 10% of the items were not completed, the sample was removed and replaced by another person.

Sampling

The following formula has been used to calculate the sample size for correlation studies:

 C = 0.5 * ln[(1+r)/(1-r)] = 0.2059,

α (two-tailed) = 0.05 - threshold probability for rejecting the null hypothesis (type I error rate)

β = 0.20 - probability of failing to reject the null hypothesis under the alternative hypothesis (type II error rate)

r = 0.203 - the expected correlation coefficient

The standard normal deviate for α = Zα = 1.9600

The standard normal deviate for β = Zβ = 0.8416

Total sample size = N = [(Zα+Zβ)/C]2 + 3 = 188

Considering the correlation coefficient of 0.203 power 80% and the estimation error of 0.05%, as well as considering 10% of possible shedding of samples, the sample size was estimated to be 212. We increased the sample size to 220 people. Using a multi-stage cluster sampling method, 10 health centers were chosen out of 21 health centers in Hamadan City (50% of clusters). After making the necessary arrangements with the health centers, a list of pregnant women aged 28-38 weeks was prepared using the existing data recorded in the Integrated Health System of health centers (about 20-25 people in each center). By coordination with the midwives responsible for providing prenatal care services to the identified women, pregnant women who met the inclusion criteria were invited for interview by phone. Sampling was done by the study's first author, who had acquired the necessary skills for sampling at the student research center. After explaining the research purpose, written consent was obtained, and the participants completed the questionnaires using the self-report method.

Data collection instruments

Pittsburgh Sleep Quality Index (PSQI)

The questionnaire has a total score of 0 to 21, which is calculated by the sum of mean scores of seven subscales, namely, subjective sleep quality, sleep latency, sleep duration, sleep efficiency, sleep disturbance, use of sleep medication, and daytime dysfunction. Good sleep quality is having a PSQI score <5, and individuals with a PSQI score >5 were said to have poor sleep quality. The validity and reliability of the Persian version of the PSQI have been confirmed in Iran [19].

Prenatal Distress Questionnaire (PDQ)

The Prenatal Distress Questionnaire (PDQ) was developed in the United States to assess specific worries and concerns during pregnancy. The questionnaire has 12 Items and three subscales, including concerns about birth and the baby, physical symptoms and body image, and emotions and relationships. The questionnaire has a total score of 12 to 60; the higher the score, the more worries [20]. The validity and reliability of the Persian version of PDQ in Iran have been confirmed [21].

Inter-item reliability was examined to ensure homogeneity and stability of measurements in the research setting. For this purpose, Cronbach's alpha was used. The cut-off point of 0.7 was considered for determining the internal consistency. In the present study, the reliability was calculated to equal 0.84 for the PSQI and 0.93 for the PDQ.

Demographic Characteristics Questionnaire

This includes the age, occupation, and education level of women and their spouses, location, gestational age, prenatal care, and pregnancy situation.

Ethical statement

This study was conducted in accordance with the declaration of Helsinki. The study was approved by the Ethics Committee of the Research Council of Hamadan University of Medical Sciences (IR.UMSHA.REC.1399.248). Written consent was obtained from the participants, and they were assured of the confidentiality of their information.

Statistical analysis

In addition to descriptive analysis, the Pearson correlation coefficient and independent t-test were used to analyze the relationships between variables. Finally, univariate and multivariate regression logistic models with the backward method were performed at a 95% confidence level.

## Results

The participants’ mean age was 27.01 ± 6.41 years, and their spouses’ mean age was 31.60 ± 5.79 years. In addition, the participants’ mean gestational age was 32.56 ± 3.5 weeks. Table 1 shows the other demographics of the participants.

**Table 1:** Sociodemographic characteristics of the participants (n = 220) *All data are indicated in number (percent).

Variable	N (%)^*^	Variable	N (%)^*^	Variable	N (%)^*^
Educational level		Husband’s educational level		Location	
Under diploma	106 (48.2)	Under diploma	82 (37.3)	Urban	135 (61.4)
Diploma	55 (25)	Diploma	95 (43.2)	Rural	85 (38.6)
Associate degree	31 (14.1)	Associate degree	24 (10.9)	Prenatal care	
Bachelor’s degree	24 (10.9)	Bachelor’s degree	17 (7.7)	Regular	209 (95)
Master’s degree	4 (1.8)	Master’s degree	2 (0.9)	Irregular	11 (5)
Occupation		Husband’s occupation		Pregnancy situation	
Housewife	171 (77.7)	Clerk	75 (34.1)	Wanted	190 (86.4)
Employed	49 (22.3)	Shopkeeper	137 (62.3)	Unwanted	30 (13.6)
		Others	8 (3.7)		

The pregnant women’s total sleep quality score was 13.8 ± 3.08, indicating that the pregnant women had poor sleep quality. According to PSQI ≥ 5 as a cut-off point, 76.3% of the study’s participants had poor sleep quality. On the other hand, the total pregnancy concerns score was 36.02 ± 6.83, indicating that the pregnant women’s concerns were moderate (Table 2).

**Table 2:** Descriptive analysis of the pregnancy concerns and sleep quality and their subscales in the pregnant women (n = 220) ^a^ PDQ (Prenatal Distress Questionnaire); ^b^ PSQI (Pittsburgh Sleep Quality Index); ^c^ Mean (standard deviation); ^d^ Median (25th and 75th percentiles); ^e^ Minimum; ^f^ Maximum

Variables	Mean (SD)^a^	Med (P25%-P75%)^ b^	Min^c^	Max^d^
Total PDQ score ^a^	36.02 (6.83)	36.50 (32-40)	18	53
Concerns about birth and the baby	16.60 (3.08)	17 (15-19)	9	24
Concerns about emotions and relationships	9.89 (3.52)	10 (8-12)	1	19
Concerns about physical symptoms and body image	9.51 (2.79)	9 (8-12)	3	18
Total PSQ score^ b^	13.08 (3.08)	8 (6-10)	1	17
Sleep latency	1.59 (0.75)	2 (1-2)	0	3
Sleep duration	0.93 (1.05)	1 (0-3)	0	3
Subjective sleep quality	1.36 (0.76)	1 (1-2)	0	3
Sleep efficiency	1.58 (1.33)	2 (0-3)	0	3
Sleep disturbance	1.43 (0.66)	1 (1-2)	0	3
Use of sleep medication	0.39 (0.65)	0 (0-1)	0	3
Daytime dysfunction	0.83 (0.79)	1 (0-1)	0	3

According to the Pearson correlation test, the total score of pregnancy concerns and its subscales were correlated with sleep quality directly (P < 0.001), which means that women with higher concern scores had lower sleep quality. There was also a statistically significant inverse correlation between age and sleep quality; the younger people had more sleep disorders (P = 0.03). However, the correlation between gestational age and sleep quality was not significant (P = 0. 26)(Table 3).

**Table 3:** Relationship between the total PSQI score with the total PDQ score and its subscales, age, and gestational age of pregnant women *Pearson test; PDQ (Prenatal Distress Questionnaire); PSQI (Pittsburgh Sleep Quality Index)

Variables	r	P*
Concerns about birth and the baby	0.207	0.002
Concerns about physical symptoms and body image	0.281	0.000
Concerns about emotions and relationships	0.411	0.000
Total PDQ score	0.419	0.000
Age	-0.13	0.03
Gestational age	0.075	0.26

According to the independent t-test results, none of the variables of the education level of women and their spouses, location, gestational age, prenatal care, and pregnancy situation were related to sleep quality. Using logistic regression (backward LR) for determining the relationship between the total PDQ score and sociodemographic characteristics with the total PSQI score, the variables (age, husband’s age, educational level, husband’s educational level, occupation, location, pregnancy situation, prenatal care, gestational age, and total pregnancy concerns score) entered the model. An unadjusted general linear model found a significant relationship between the total PDQ score, age, husband’s age, husband’s educational level, occupation, and total PSQI score. The confounding variables exclude step by step. It was done in six steps, and the final model showed a significant relationship between the total PDQ score and two variables of educational level (diploma) and husband’s educational level (under diploma) with the total PSQI score (Table 4).

**Table 4:** Relationship between the total PDQ score and sociodemographic characteristics with the total PSQI score in pregnant women based on the general linear model (n = 220) PDQ: Prenatal Distress Questionnaire; PSQI: Pittsburgh Sleep Quality Index *Academic indicates Bachelor’s degree and Master’s degree. **Adjusted for educational level, location, pregnancy situation, prenatal care, and gestational age; R^2^= 0.251.

Variables	Univariate regression (Unadjusted)		Multivariate regression (adjusted)^**^	
	P-value	β [95% CI]	P-value	β [95% CI]
Occupation (housewife)				
Employed	0.09	0.79 [0.53, 1.7]	0.007	1.32 [0.36, 2.28]
Educational level (academic*)				
Below diploma	0.45	0.37 [0.59, 1.34]	0.30	0.05 [1.47, 0.45]
Diploma	0.01	1.19 [0.19, 2.18]	0.18	0.75 [0.35, 1.87]
Location (rural)				
Urban	0.62	-0.18 [-0.92, 0.55]	0.42	-0.34 [-1.17, 0.49]
Pregnancy situation (unwanted)				
Wanted	0.90	0.06 [-0.99, 1.12]	0.74	-0.19 [-1.37, 0.98]
Prenatal care (irregular)				
Regular	0.94	0.05 [-1.62, 1.73]	0.39	0.80 [-1.05, 2.66]
Husband’s educational level (academic ^a^)				
Under diploma	0.04	1.03 [0.02, 2.03]	0.01	1.47 [2.59, -0.35]
Diploma	0.54	0.30 [0.68, 1.30]	0.12	0.84 [1.93, 0.24]
Age	0.54	-0.02 [-0.11, 0.05]	0.03	-0.06 [-0.13, -0.004]
Husband’s age	0.22	-0.06 [-0.15, 0.03]	0.000	-0.12 [-0.19, -0.05]
Gestational age	0.26	0.06 [-0.04, 0.17]	0.26	0.06 [-0.04 , 0.18]
Total pregnancy concerns score	0.000	0.17 [0.11, 0.22]	0.000	0.18 [0.13-0.24]

## Discussion

This study predicted sleep quality in primigravid women based on pregnancy concerns. Pregnant women's PSQI score was 10.8 (3.08). According to PSQI ≥ 5, which is considered poor sleep quality, 76.3% of our study's participants had poor sleep quality. We found that pregnancy concerns, including concerns about birth and the baby, concerns about physical symptoms and body image, and concerns about emotions and relationships, can reduce the sleep quality of primigravid women. Various studies have found a significant increase in the rate of sleep disorders in pregnant women [22-23]. Using data from 8798 participants, Salari et al. conducted a systematic review and meta-analysis study to evaluate the prevalence of insomnia in pregnancy. They reported that 42.4% of pregnant women have insomnia in the third trimester of pregnancy [24]. In Jemere et al.'s study on 411 Ethiopian pregnant women, more than two-thirds of pregnant women had poor sleep quality [22]. Takelle et al.'s cross-sectional study on 415 Ethiopian pregnant women showed that 42.2% of pregnant women have poor sleep quality [6]. In a meta-analysis study, Sedov et al. found that the mean PSQI score in pregnancy was 6.07 with 95% confidence (5.30, 6.85), and 45.7% of pregnant women experienced poor sleep quality [23]. Effati-Daryani et al., in a study on 605 pregnant women in Tabriz, determined the fatigue status in different trimesters of pregnancy and its relationship with sleep quality. They reported a sleep quality score of 6.56 (3.24), indicating that their study participants had lower sleep quality than ours [25]. In addition, the study of Ahmadinejad et al. was conducted to assess the sleep quality of 168 eligible women referred to health centers in Mashhad, Iran. The results showed that the average sleep quality in the third trimester was 8.27±2.91, and 89.8% of pregnant women had poor sleep quality. The authors reported a significant correlation between maternal age, poor sleep quality, and physical activity [21].

Poor sleep quality of pregnant women participating in the present study may be related to limiting the study to primigravid women and also limiting study time to the third trimester of pregnancy, where factors, such as approaching the time of childbirth, fear, and anxiety about child care and accepting the role of mother for the first time, can reduce the quality of sleep in a pregnant woman [26]. In this regard, some researchers limit the incidence of sleep disorders to the third trimester [3]. In Takelle et al.’s study, pregnant Ethiopian women in the first and third trimesters had a higher probability of having poor sleep quality than pregnant women in the second trimester [6]. In Iran, Salari et al., in their meta-analysis study using data from 8798 participants, reported that the prevalence of insomnia in the third trimester of pregnancy was 42.4% [24]. A systematic search of both international and Chinese databases by Yang et al. showed that the correlation between sleep and the mental status of pregnant women in the second trimester is more potent than in the first trimester [27]. Moreover, in a recent cross-sectional study in Iran, the prevalence rates of poor sleep quality in first, second, and third trimesters were 36%, 54%, and 62%, respectively [11]. These results suggest that all pregnant women should be screened for poor sleep quality during the third trimester.

The present study found a direct association between pregnancy concerns (PDQ score) and sleep quality (PSQI score), such that women with more concerns reported poorer sleep quality. It should be noted that although the pregnancy concerns in our pregnant women studied were at a moderate level, its relationship with sleep quality was significant. Fathi et al. examined the relationship between maternal concerns and pregnant women's quality of life and general health in Tabriz, Iran. According to their study, prenatal concerns can predict a pregnant woman's general health [16]. The results of a study by Polo-Kantola et al. on Finnish pregnant women showed that symptoms of anxiety and depression were associated with sleep problems [4]. It should be noted that although the pregnancy concerns in our pregnant women studied were at a moderate level, its relationship with sleep quality was significant. The sleep quality and associated factors of 78 healthy Finnish pregnant women were studied by Polo-Kantola et al. The results showed that poor general sleep quality, difficulty falling asleep, the number of nocturnal awakenings per night, and too-early morning awakenings increased in late pregnancy compared with mid-pregnancy. In addition, symptoms of anxiety and depression were associated with sleep problems [4].

According to the Pearson test, there was a significant inverse correlation between the age of the participants in our study and their sleep quality, meaning that the younger the people were, the more sleep disorders they had. Gao et al.’s study in China found an association between poor sleep quality and postpartum depression in women more than or equal to 30 years old. However, this relationship was insignificant in women under 30 [10]. On the other hand, sleep quality has decreased with age in some studies. For example, in a study in Mashhad, increasing maternal age was associated with poor sleep quality 30 [21]. Higher maternal age, higher gestational age, and multiparity were also predictors of poor sleep quality in pregnancy in Jemere et al.’s study [22]. In addition, some studies have not found a significant relationship between maternal age and sleep quality [20].

In the present study, educational level was the predictor of sleep quality, and less education was associated with poorer sleep quality. However, due to the study's cross-sectional design, the causal relationships between educational level and sleep quality could not be assessed. In line with the present study, Dabiran and colleagues, in a cross-sectional study on 400 pregnant women in Tehran, Iran, reported better sleep quality in women with higher levels of education [28]. Jahdi et al.'s study showed that sleep disorders were less common in highly educated people [20]. Several studies have been conducted to understand how education affects well-being. For instance, education has directly influenced happiness [29]. Shim et al.'s study confirmed that the moderated effect of the education level was related to the effect of sleep quality, which showed a moderating effect on the effect of depression on suicidal ideations. Health behaviors, social interactions, and income were examined as potential mechanisms to mediate the effects of education on well-being [30]. In contrast with our finding, the study of Azarniveh et al. reported no relationship between job and education variables and sleep quality [26].

This study has produced evidence about the relationship between pregnancy concerns and sleep quality. However, the causal relationships among the variables could not be assessed due to the study's cross-sectional design. One of the main limitations of the present study was the small sample size of the participants. In addition, a wide confidence interval was noted in some variables due to the small sample size. Further studies with a larger sample size from different settings are needed to increase the accuracy and generalizability of the findings. Furthermore, factors affecting the adaptation of pregnant women to pregnancy concerns, as well as the significant factors affecting sleep quality, have not been considered in this study.

## Conclusions

The present study shows that the prevalence of poor sleep quality among pregnant women was high. Considering the adverse effects of poor sleep quality on pregnancy outcomes, incorporating the screening program of pregnant women in terms of sleep quality into routine prenatal care services is recommended. The study revealed that pregnancy concerns, including concerns about birth and the baby, concerns about physical symptoms and body image, as well as concerns about emotions and relationships, can reduce the sleep quality of primigravid women. Designing and implementing effective interventions to reduce or modify these common concerns can improve the sleep quality of pregnant women and prevent the adverse effects of poor sleep quality on pregnancy outcomes.
